# Immune Infiltration, Cancer Stemness, and Targeted Therapy in Gastrointestinal Stromal Tumor

**DOI:** 10.3389/fimmu.2021.691713

**Published:** 2021-12-03

**Authors:** Jingjing Wang, Hui Ren, Wenhui Wu, Qianlin Zeng, Jingyao Chen, Juanjuan Han, Minquan Lin, Changhua Zhang, Yulong He, Mingzhe Li

**Affiliations:** ^1^ Department of Laboratory, Hexian Memorial Hospital of Panyu District, Guangzhou, China; ^2^ Digestive Medicine Center, The Seventh Affiliated Hospital, Sun Yat-sen University, Shenzhen, China

**Keywords:** gastrointestinal stromal tumor, targeted therapy, immune infiltration, cancer stem cells, tumor immune microenvironment

## Abstract

**Objective:**

To investigate the characteristics of the tumor immune microenvironment in patients with gastrointestinal stromal tumor (GIST) and identify cancer stem-like properties of GIST to screen potential druggable molecular targets.

**Methods:**

The gene expression data of 60 patients with GIST was retrieved from the Array Express database. CIBERSORT was applied to calculate the level of immune infiltration. ssGSEA and ESTIMATE were used to calculate the cancer stemness index and tissue purity. The Connectivity Map (CMAP) database was implemented to screen targeted drugs based on cancer stem-like properties of GIST.

**Result:**

There was a difference in the level of immune infiltration between the metastasis and non-metastasis GIST groups. The low level of T-cell infiltration was correlated with high tumor purity and tumor stemness index, and the correlation coefficients were -0.87 and -0.61 (p < 0.001), respectively. Furthermore, there was a positive correlation between cancer stemness index and cell purity (p < 0.001). The cancer stemness index in the metastasis group was higher than that in the non-metastasis group (p = 0.0017). After adjusting for tumor purity, there was no significant correlation between T-cell infiltration and cancer stemness index (p = 0.086). Through the pharmacological mechanism of topoisomerase inhibitors, six molecular complexes may be the targets of GIST treatment.

**Conclusion:**

Immune infiltration in GIST patients is related to cancer stem-like properties, and the correlation relies on tumor purity. Cancer stemness index can be used as a new predictive biomarker of tumor metastasis and targets of drug therapy for GIST patients.

## Introduction

Gastrointestinal stromal tumor (GIST) is the most common gastrointestinal mesenchymal tumor. The common sites of GIST are the stomach, intestine, colon, etc. It originates from interstitial cells of Cajal (ICC) or more primitive progenitor cells (1). There are functional mutations in GIST due to acquisition of c-kit gene (75%–80%) or PDGFRA gene (5%–10%) (2–6). These mutations can lead to the continuous activation of ligand-independent receptor proteins, and then downstream signal transduction pathways being activated, and afterward cell apoptosis being inhibited and cell proliferation being promoted, which is considered to be the classic pathogenesis of GIST. It was reported that all GIST patients represented malignant behavior, with features of invasion, and metastasis.

The relationship between cancer stem-like properties and immunity has become one of the hotpots recently, because of the development of immunotherapy. On the other hand, a series of studies has focused on the relationship between immune infiltration and prognosis of cancer patients (7, 8). Cancer cells with stem-like properties (9–11) are responsible for tumor recurrence, metastasis, drug resistance, and other prognostic effects (12–15). Emerging evidence suggests that metastasis and recurrence of GIST may be closely related to the cancer cells with stem-like properties (16). Thus, disclosure of the characteristics of the tumor immune microenvironment and cancer stem-like properties of GIST patients are important for improving tumor treatment; however, these are still unmet medical needs. To achieve such targets, the molecular mechanisms that determine how cancer cells with stem-like properties are involved in the pathogenesis of GIST need to be elucidated.

In this study, the CIBERSORT algorithm was applied to calculate the level and characteristics of immune infiltration in GIST patients. GO analysis and enrichment of KEGG pathway were conducted based on the differential genes identified by combining the gene expression profiles of stem-like cancer cells and cell purity. Then, PPI network analysis and characteristic module of the differential genes were carried out. Finally, specific novel molecules targeting the cancer stem-like properties of GIST were analyzed by the CMAP database to achieve the purpose of providing information for developing targeted drugs. Therefore, our research could provide not only a scientific basis for the pathogenesis of GIST but also scientific evidence for the development of targeted medicine.

## Materials and Methods

### Data Collection and Processing

Gastrointestinal stromal tumor (GIST) RNA-seq gene expression data and patient clinical data were downloaded from the Array Express database (https://www.ebi.ac.uk/arrayexpress/; chip number: E-MTAB-373; name: transcription profiling by array of human GIST to validate prognostic signature). Sixty GIST patients, including 15 metastatic cases and 45 non-metastatic cases, were enrolled. The chip type was the Agilent single channel. We downloaded the original data by using R package “ArrayExpress.” For gene expression array data, background correction was carried out using the “backgroundCorrect” function of the R package “limma” with default parameters.

### Analyze Gene Expression Data

CIBERSORT algorithm: CIBERSORT is a tool used for deconvolution of the expression matrix of immune cell subtypes based on the principle of linear support vector regression. We could get the infiltrating information of 22 kinds of immune cells from GIST sequencing data calculated by the CIBERSORT algorithm, with the support of the web tool (https://cibersort.stanford.edu/).

The expression of EMT (epithelial–mesenchymal transition, EMT) related genes: the EMT-related gene expression signature was comprised of 200 genes obtained from gene set “hallmark epithelial mesenchymal transition” in The Molecular Signatures Database (MSigDB, https://www.gsea-msigdb.org/gsea/msigdb). For each sample in the Array Express database, an EMT expression value was calculated by the arithmetic mean of these 200 EMT gene expression levels (in the log2 scale). Similarly, the expression value of characteristic genes of T cells was calculated by this method and the correlation between them was also analyzed.

T-cell infiltration fraction: to derive T-cell markers, we downloaded the gene expression profiles of 513 cell-type markers across 22 different types and states of immune cells used by CIBERSORT. Genes with standardized (Z-score) expression value ≥ 2 in at least one T-cell subtype/status were considered T-cell markers resulting in a set of 156 T-cell markers. After removing genes that did not exist in the expression data, a total of 143 T-cell markers were used for subsequent analysis. The level of T-cell infiltration was estimated by the arithmetic mean of the 143 T-cell marker expression levels (in the log2 scale). In order to compare and analyze the difference between T-cell infiltration and cancer stem-like properties, we used the ssGSEA algorithm to calculate the T-cell characteristic gene set fraction of each patient as T-cell infiltration fraction. The ssGSEA method was implemented using the ssGSEA function in the R package “GSVA.”

Cancer stemness index (mRNAsi): in this study, we evaluated the stemness index mRNAsi by using the GSVA package in R (17). The log2-transformed normalized values of gene expression data were used to generate the mRNAsi. We obtained 109 stem cell core genes from the literature (18) and used the ssGSEA algorithm to calculate the score of stem cell core gene set of each patient as the cancer stemness index.

ESTIMATE algorithm: an algorithm that uses the ssGSEA algorithm to calculate the proportion of immune cells and matrix components in tumor tissue and evaluates the purity of tumor according to the respective composition of immune cells and stromal cells. The calculation was performed using the R package ESTIMATE.

Pathway enrichment analyses: according to cancer stemness index and tumor purity, the enrolled 60 patients were divided into two groups: high stemness index group vs. low stemness index group, high purity group vs. low purity group. The expression data were analyzed by R package “limma.” The differentially expressed genes of two groups were screened according to the standard of | log2fc | ≥ 1, p < 0.05. After the combination of the differentially expressed genes, a total of 219 genes were taken as interesting genes; disease enrichment, GO analysis, and KEGG analysis were performed using R packet “clusterProfiler.”

PPI interaction network and characteristic molecular analysis: 219 differential genes were put into the string to get PPI network. The module analysis of the PPI interaction network was performed using the MCODE tool of Cytoscape software, and the characteristic molecules were selected by the cytoHubba tool.

CMAP analysis: we employed the CMAP, a data-driven, systematic approach for discovering correlations among genes, chemicals, and biological conditions, to search for candidate compounds that might target pathways correlated with GIST stemness. Using web tools (https://clue.io/), CMAP analysis and molecular pharmacological mechanism analysis of characteristic genes can be finished.

### Statistical Analyses

R software version 3.6 (R Core Team, R Foundation for Statistical Computing, Vienna, Austria) was used for all statistical analyses. The correlation analysis was conducted by Person correlation analysis. T test or Wilcoxon test was used for continuous variables. The usage of R package was shown above. p < 0.05 was considered to be statistically significant.

## Results

### Characteristics of Immune Infiltration in GIST

We downloaded the gene expression chip data and clinical information of 60 GIST patients from the Array Express database, which consisted of 15 metastatic patients and 45 non-metastatic patients. In order to investigate the immune infiltration of GIST, the CIBERSORT algorithm was employed to calculate the expression profiles of 22 kinds of immune cells.

**Figure 1:**
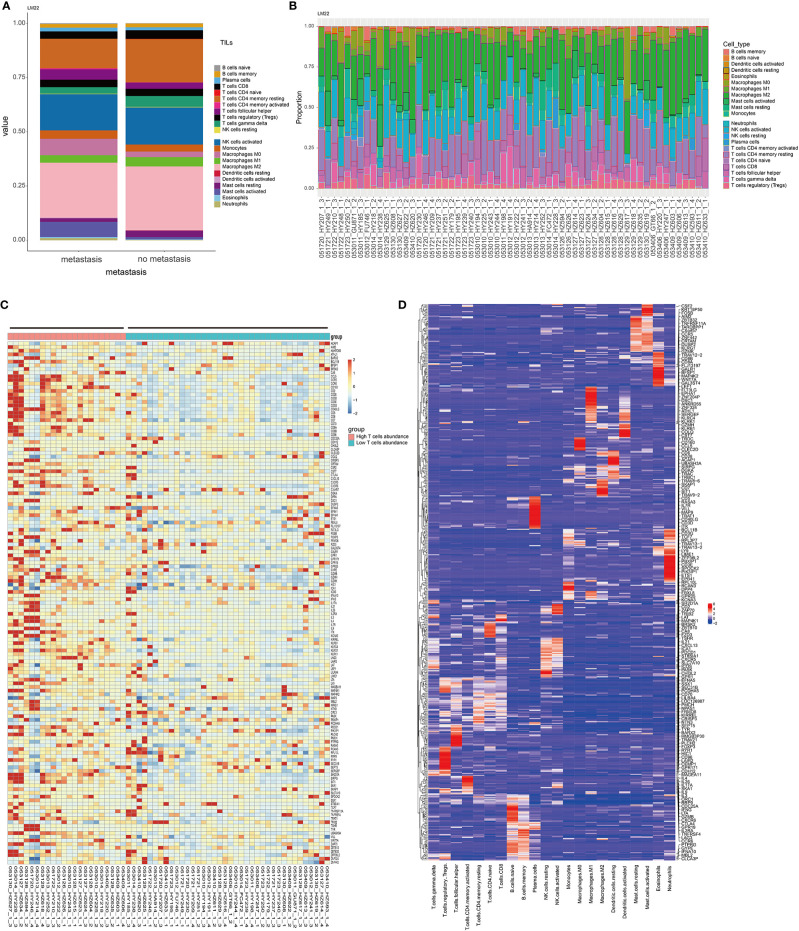
Characteristics of immune infiltration in metastatic and non-metastatic GIST. **(A)** CIBERSORT algorithm was used to calculate the difference of the infiltration ratio of 22 immune cells between the metastasis group and non-metastasis group. **(B)** The characteristics of immunocyte infiltration in 60 patients were analyzed. **(C)** Thermogram of characteristic gene expression in T cells. **(D)** Thermogram of 22 characteristic genes of immune cells. The T cell-related characteristic gene set was selected.

**Figure 2:**
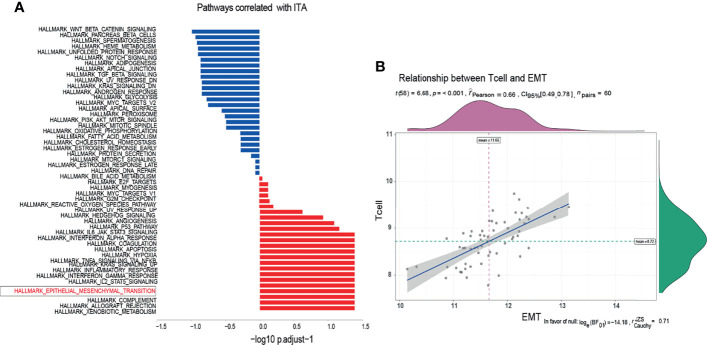
Enrichment of T-cell infiltration-related pathways. **(A)** According to the enrichment of characteristic molecules, the pathways closely related to T-cell infiltration were obtained. **(B)** The expression level of EMT characteristic molecules was positively correlated with that of T cells.

There was a significant difference in the level of immune infiltration between the metastasis group and the non-metastasis group (**Figure 1A**). For example, the M2 macrophages in the metastatic group were significantly lower than that in the non-metastatic group, indicating a higher inflammatory status in the primary site of the metastatic group. The histogram of tumor immune infiltration of the enrolled 60 patients is shown in **Figure 1B**. In order to further disclose the effect of immune infiltration, we performed gene enrichment analysis on the genes identified from the expression profile of 22 kinds of immune cells. A total of 143 marker genes related to T cells was verified (**Figure 1D**). Based on the expression level of 143 T-cell marker genes, the enrolled 60 patients were divided into high T-cell infiltration group and low T-cell infiltration group (**Figure 1C**). Afterward, pathway enrichment analysis of the genes differentially expressed in the two different T-cell infiltration groups were performed, which found that immune signaling pathways such as TNF-α/NF-kB pathway and IL2 pathway (**Figure 2A**), especially the epithelial-to-mesenchymal transition (EMT) pathway, were closely related to tumor-infiltrating T-cell abundance (ITA). Besides, we found that the level of EMT was positively correlated with the level of ITA (p < 0.001) (**Figure 2B**).

### ITA in GIST Was Negatively Correlated With the Stemness Index, and the Correlation Depended on Tumor Purity

**Figure 3:**
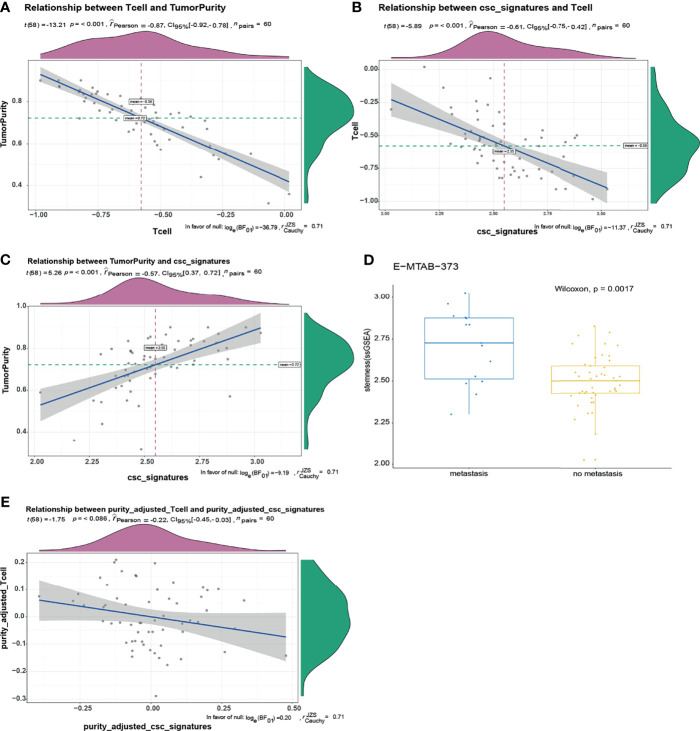
There was a negative correlation between T-cell infiltration and stemness index in GIST, and the relationship depends on tumor purity. **(A)** There was a negative correlation between tumor purity and T-cell infiltration, the correlation coefficient was -0.87, p < 0.001. **(B)** T-cell infiltration was negatively correlated with cancer stemness index. The correlation coefficient was -0.61, p < 0.00. **(C)** There was a positive correlation between tumor purity and cancer stemness index. The correlation coefficient was 0.57, p < 0.001. **(D)** The cancer stemness index of tumor metastasis group was significantly higher than that of non-metastasis group (p = 0.0017). **(E)** After adjusting for tumor purity, there was no significant correlation between T-cell infiltration and cancer stemness index.

We explored the relationship between ITA and cancer stem-like properties. One hundred nine core genes related to cancer stem-like properties were selected. Single-sample gene enrichment analysis (ssGSEA) was used to calculate the cancer stemness index, and the ssGSEA value of core genes of T cells was calculated as the index of ITA. The tumor purity was calculated using the ESTIMATER package by calculating and analyzing the microarray expression data. ESTIMATER could distinguish the two gene expression profiles to identify immune cells and stromal cells (immune-ESTIMATE and stromal-ESTIMATE). The purity of tumor could be evaluated based on the composition of immune cells and stromal cells. The results showed that a low level of ITA was associated with higher tumor purity and cancer stemness index, with correlation coefficients of -0.87 and - 0.61, respectively (p < 0.001) (**Figures 3A, B**). Besides, there was a positive correlation between cancer stemness index and tumor purity, and the correlation coefficient was 0.57 (p < 0.001) (**Figure 3C**). The cancer stemness index in the metastasis group was higher than that in the non-metastasis group (p = 0.0017) (**Figure 3D**). After adjusting for tumor purity, there was no significant correlation between ITA and cancer stemness index (p = 0.086) (**Figure 3E**). These results indicated that the correlation between ITA and the cancer stemness index depended on tumor purity, and the cancer stemness index in the GIST metastasis group was higher than that in the non-metastasis group. It also suggested that GIST patients with metastasis have more abundant cancer stem-like properties and more serious tumor resistance.

### Differential Gene Analysis Based on Cancer Stem-Like Properties and Tumor Purity

**Figure 4:**
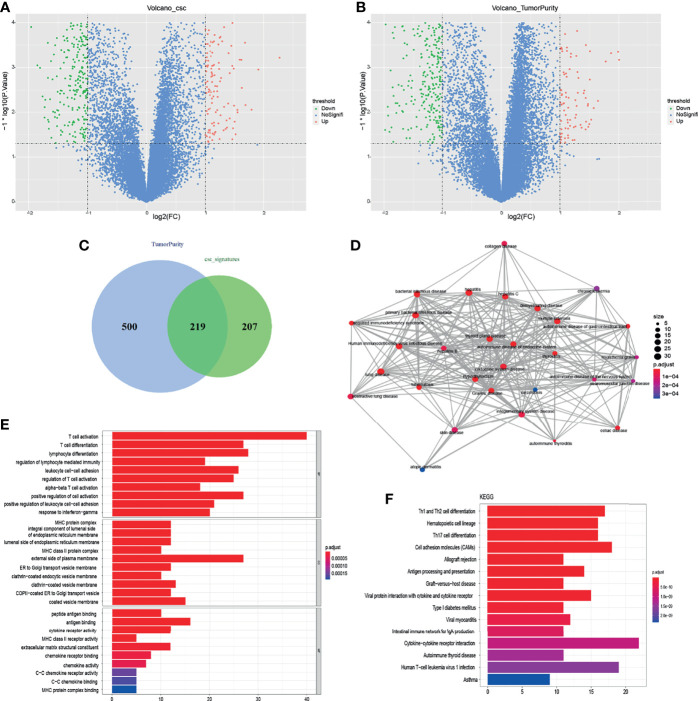
Analysis of gene differences by combining cancer stemness index with tumor purity. **(A)** Gene volcano map of cancer stemness index difference. **(B)** Gene volcano map of tumor purity difference. **(C)** Two hundred nineteen differentially expressed genes were identified by the analysis of cancer stem-like properties and purity. **(D)** According to GO analysis, 219 gene diseases were enriched to obtain diseases significantly related to different genes. **(E, F)** GO analysis and KEGG analysis, respectively.

In order to explore the difference of gene expression between cancer stem-like properties and tumor purity, we analyzed the gene expression data of the enrolled 60 patients derived from E-MTAB-373 (**Figures 4A, B**). Two hundred nineteen differential genes were obtained based on the filter criteria of p < 0.05 and | log2fc | > 1. According to the level of the cancer stemness index, the patients were divided into high and low index groups, and 426 differential genes were recognized (**Figure 4A**). According to the level of tumor purity, the patients were divided into high-purity and low-purity groups, and 719 differential genes were obtained (**Figure 4B**). Two hundred nineteen differentially expressed genes were obtained by combining two sets of differentially expressed genes (**Figure 4C**). Then, we used pathway enrichment analysis to analyze 219 differentially expressed genes. The results indicated that endocrine autoimmune system diseases such as Graves’ disease and immune system diseases were enriched, which proved that cancer stem-like properties were closely related to autoimmune system diseases (**Figure 4D**). The results of GO analysis showed that T cells were activated and differentiated, and the autoimmune-specific antigen receptors were enriched (**Figure 4E**). In addition, Th1, Th2, and Th7 cell differentiation pathways and cell adhesion pathways were enriched (**Figure 4F**), which further indicated that cancer stem-like properties were closely related to immune infiltration.

### PPI Network and Module Analysis

**Figure 5:**
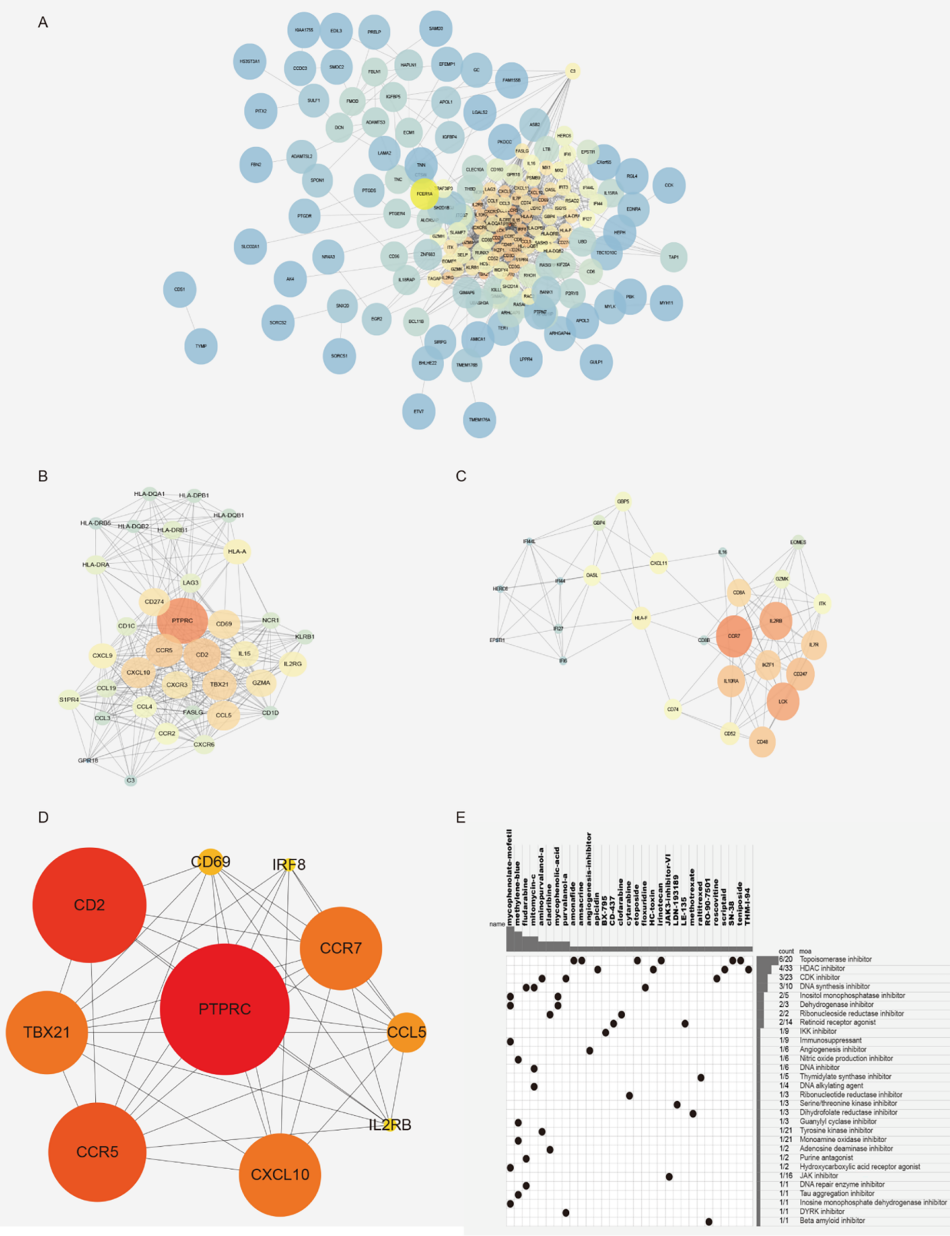
Gene module analysis and characteristic molecular screening. **(A)** PPI network diagram, 219 difference interaction diagrams were obtained by using the String website. **(B, C)** Characteristic module analysis. **(D)** The top 10 characteristic molecules were obtained by the screening of characteristic molecules. **(E)** CMAP mode of action analysis (MoA) was used analyze the molecular pharmacological mechanism enrichment of related inhibitors.

In order to understand the relationship and function of the 219 differential genes we screened, the String website was applied to predict the interaction of the 219 genes. The results are shown in **Figure 5A**. Then, the key gene (hub gene) screening and functional module analysis were carried out by using the software of Cytoscape. Eleven functional modules were obtained, and the two modules with the highest scores are shown in **Figures 5B, C**. According to the type of molecules in the module, it could be found that the module was closely related to the immune system. To further identify the characteristic molecules, we analyzed the 219 genes using the cytoHubba and selected the top 10 genes (PTPRC, CD2, CD69, IRF8, CCR7, CCL5, il2rb, CXCL10, CCR5, TBX21) as new molecular markers of cancer stem-like cells for GIST patients (**Figure 5D**). According to PPI network and module analysis, the cancer stem-like properties were closely related to the immune system.

### CMAP Analyses Identified Novel Candidate Compounds Targeting Cancer Stem-Like Properties

In order to identify novel compounds with cancer stem-like properties, 219 differentially expressed genes were divided into high and low stemness index groups according to the value of log2FC from CMAP analysis. Thirty-four molecular pharmacological mechanisms were obtained by CMAP mode of action analysis (MoA) of 23 complexes with correlation fraction ≤-90 (**Figure 5E**). Among them, the pharmacological mechanism of topoisomerase inhibitors was enriched in six compounds: amonafide, irinotecan, SN-38, etoposide, amsacrine, and teniposide. Four compounds (apicidin, HC toxin, scriptaid, and thm-i-94) were HDAC inhibitors and three compounds (purvalanol-a, aminopurvalanol-a, and roscovitine) were CDK inhibitors. New drug molecular complexes might specifically inhibit the proliferation and division of cancer cells.

## Discussion

GIST is the most common gastrointestinal tumor derived from mesenchymal tissue. In recent years, 80% of GIST patients can be clinically cured by radical resection combined with imatinib adjuvant therapy. However, the prognosis of some GIST patients remains unfavorable due to cancer metastasis, recurrence, and drug resistance. Previous studies had demonstrated that immunotherapy had a positive effect on GIST, but there were few studies that focused on tumor microenvironment and immunotherapy of GIST. In this study, the CIBERSORT algorithm was used to calculate the proportion of infiltrated immune cells in GIST tissues to determine the pattern of immune infiltration in metastatic and non-metastatic tumor tissues (19, 20). We found that there were many differences in the composition of immune cells in the metastatic group compared with the non-metastatic group. Studies had shown that there were a large number of tumor-infiltrating T-cells in GIST. According to this idea, GIST patients were divided into high ITA group and low ITA group. The results showed that the EMT signaling pathway was significantly positively correlated with lymph node invasion, suggesting that the local immune microenvironment may be related to tumor metastasis.

Recent studies had found that cancer stem-like properties played a very important role in the metastasis, recurrence, and drug resistance of solid tumors. Studies in GIST had also confirmed that cancer stem-like properties were closely related to drug resistance (16). We used the ssGSEA algorithm to calculate the enrichment fraction of the core gene set of cancer stem-like properties as the cancer stemness index (18, 21, 22) and compared the difference of the cancer stemness index between the metastasis group and non-metastasis group. The result showed that the cancer stemness index in the metastasis group was significantly higher than that in the non-metastasis group, indicating that the poor treatment effect in the metastasis group may be related to the high cancer stemness index. It was also suggested that different clinical treatments should be explored for GIST patients with or without metastasis.

A unified algorithm was used to calculate the relationship between cancer stemness index and tumor immune infiltration (23). We found that there was a negative correlation between tumor immune infiltration and cancer stemness index. Moreover, the relationship depended on tumor purity. The higher the purity, the higher the cancer stemness index. This trend was consistent with the characteristics of tumor derived from mesenchymal tissue (24). We also found a weak correlation between cancer stemness index and tumor EMT level, indicating that there was a certain difference between mesenchymal and epithelial tumors.

Finally, we identified the interesting genes according to the differential genes based on the two groups of different cancer stemness index and two groups of different tumor purity. Then, pathway analysis was conducted, which found that the pathways related closely to the tumor immune system, proving the close relationship between tumor immune microenvironment and cancer stem-like properties. In the process of module analysis and characteristic molecular screening, we selected two characteristic modules and 10 characteristic molecules (PTPRC, CD2, CD69, IRF8, CCR7, CCL5, il2rb, CXCL10, CCR5, TBX21), which could be used as biomarkers of cancer stem-like cells for GIST patients. Finally, we screened these characteristic molecules and identified some novel candidate compounds that targeted the stem-like properties of GIST patients.

## Conclusions

In conclusion, the immune microenvironment in metastatic and non-metastatic GIST tissues was quite different. The level of tumor immune infiltration was closely related to cancer stem-like properties. The level of cancer stemness index in the metastasis tumor was significantly higher than that of non-metastasis. Through a series of characteristic molecular screening, we had identified the characteristic molecules closely related to cancer stem-like properties and further identified novel candidate compounds targeting the GIST stem-like properties. In the future, we will verify the molecular pathways *in vivo* and *in vitro*, in order to provide scientific basis for the pathogenesis of GIST and the development of targeted drugs.

## Data Availability Statement

Publicly available datasets were analyzed in this study. This data can be found here: Gene expression data and clinical data were obtained from ArrayExpress database (https://www.ebi.ac.uk/arrayexpress/). Chip number: E-MTAB-373, Name: Transcription profiling by array of human GIST to validate prognostic signature.

## Author Contributions

JW, CZ, MzL, and YH designed the research; JW, HR, and QZ collected the experimental data. JW, JC, MqL, and JH performed the analysis. JW, CZ, MzL, and YH wrote the manuscript. All authors contributed to the article and approved the submitted version.

## Funding

This work was supported by the Science and Technology Planning Project of Guangdong Province, China (2017A020215014); the Science and Technology Planning Project of Shenzhen, China (JCYJ20190809142807444); San-ming Project of Medicine in Shenzhen, China (00101100017); the Science and Technology Planning Project of Guangzhou, China (202102080603).

## Conflict of Interest

The authors declare that the research was conducted in the absence of any commercial or financial relationships that could be construed as a potential conflict of interest.

## Publisher’s Note

All claims expressed in this article are solely those of the authors and do not necessarily represent those of their affiliated organizations, or those of the publisher, the editors and the reviewers. Any product that may be evaluated in this article, or claim that may be made by its manufacturer, is not guaranteed or endorsed by the publisher.
